# High-Quality Genome Sequence of *Bacillus vireti* DSM 15602^T^ for Setting Up Phylogenomics for the Genomic Taxonomy of *Bacillus*-Like Bacteria

**DOI:** 10.1128/genomeA.00864-15

**Published:** 2015-08-06

**Authors:** Guo-Hong Liu, Bo Liu, Jie-Ping Wang, Jian-Mei Che, Qian-Qian Chen, Zheng Chen

**Affiliations:** Agricultural Bio-Resources Research Institute, Fujian Academy of Agricultural Sciences, Fuzhou, China

## Abstract

*Bacillus vireti* DSM 15602^T^ is a Gram-negative, spore-forming, and facultatively anaerobic bacterium. Here, we report the 5.309-Mb draft genome sequence of *B. vireti* DSM 15602^T^, which will provide useful information for setting up phylogenomics for the genomic taxonomy of *Bacillus*-like bacteria, as well as for the functional gene mining and application of *B. vireti*.

## GENOME ANNOUNCEMENT

*Bacillus vireti* DSM 15602^T^ is a Gram-negative facultatively anaerobic bacterium isolated from the soil of several disused hay fields in the Netherlands (1). From 6S rRNA phylogenetic analysis, it was observed that strain DSM 15602^T^ is very closely related to *Bacillus novalis*, *Bacillus soli*, *Bacillus bataviensis*, and *Bacillus drentensis*.

At the present, there are very few studies about *B. vireti*. Mohandass et al. (2) found that the mixed culture of *Bacillus cereus* and *B. vireti* isolated from the petrochemical industry had the ability to biodegrade benzo[*a*]pyrene. Mania et al. (3) provided more detailed phenotypic characterizations of *B. vireti* with respect to its dissimilar NO_3_ metabolism and presented data from a whole-genome analysis of this organism, with special interest in aspects relating to nitrogen transformation. However, the genome sequence provided by Mania et al. (3) was low quality, with 228 contigs, which could not be used in setting up phylogenomics for the genomic taxonomy of *Bacillus*-like bacteria. To set up the genomic taxonomy system, the species of *Bacillus*-like bacteria will be sequenced one by one. In this study, a more high-quality genome sequence of *B. vireti* DSM 15602^T^ was obtained, which will promote research on the genomic taxonomy of *Bacillus*-like bacteria.

The genome sequencing of *B. vireti* DSM 15602^T^ was performed on an Illumina HiSeq 2500 system by generating paired-end libraries. Paired-end reads were *de novo* assembled using SOAP*denovo* version 1.05 (4). DNA libraries with insert sizes of 500 bp were constructed and sequenced using the 2 × 150-bp paired-end sequencing strategy. After filtering of the 1.67 Gb of raw data, the 1.56-Gb clean sequence data were obtained, providing approximately 300-fold coverage. Through the data assembly, 5,309,094 bp within 27 scaffolds were obtained, and the scaffold *N*_50_ was 530,832 bp. The average length of the scaffolds was 196,633 bp, and the longest and shortest scaffolds were 385,746 bp and 507 bp, respectively. Of the clean reads, 96.04% were aligned back to the genome, suggesting a good quality of the assembly.

Gene prediction was performed using Glimmer version 3.02 (5), and rRNAs and tRNAs were identified using RNAmmer (6) and tRNAscan-SE 1.3.1 (7), respectively. A total of 5,118 genes were predicted, including 4,794 coding sequences (CDS), 221 pseudogenes, 33 frameshifted genes, 1 noncoding RNA (ncRNA), 89 tRNAs, and 13 rRNA genes. Also, 2 clustered regularly interspaced short palindromic repeat (CRISPR) arrays were found in the draft genome. The average DNA G+C content was 39.85%, being compatible with the value of 40.2 mol% acquired by high-performance liquid chromatographic (HPLC) determination (1). Gene function was analyzed by BLAST of the amino acid sequence against the COG and KEGG databases. There were 3,592 and 2,364 genes assigning to the COG and KEGG databases, respectively.

From the genome sequence analysis, *B. vireti* DSM 15602^T^ was predicted to possess complete carbon metabolic pathways, including those for glycolysis, fatty acid biosynthesis, and the pentose phosphate pathway. In particular, *B. vireti* DSM 15602^T^ was predicted to be equipped with a wide variety of genes for amino acid and organic acid metabolism, agreeing with the physiological properties of *B. vireti* DSM 15602^T^ (1).

### Nucleotide sequence accession numbers.

This whole-genome shotgun project has been deposited at DDBJ/EMBL/GenBank under the accession no. LDNB00000000. The version described in this paper is version LDNB01000000.

## References

[1] Heyrman J, Vanparys B, Logan NA, Balcaen A, Rodríguez-Díaz M, Felske A, De Vos P 2004 *Bacillus novalis* sp. nov., *Bacillus vireti* sp. nov., *Bacillus soli* sp. nov., *Bacillus bataviensis* sp. nov. and *Bacillus drentensis* sp. nov., from the Drentse A grasslands. Int J Syst Evol Microbiol 54:47–57. doi:10.1099/ijs.0.02723-0. 14742458

[2] Mohandass R, Rout P, Jiwal S, Sasikala C 2012 Biodegradation of benzo[*a*]pyrene by the mixed culture of *Bacillus cereus* and *Bacillus vireti* isolated from the petrochemical industry. J Environ Biol 33:985–989. 23741789

[3] Mania D, Heylen K, van Spanning RJM, Frostegård A 2014 The nitrate-ammonifying and *nosZ*-carrying bacterium *Bacillus vireti* is a potent source and sink for nitric and nitrous oxide under high nitrate conditions. Environ Microbiol 16:3196–3210. doi:10.1111/1462-2920.12478. 24708037

[4] Li R, Zhu H, Ruan J, Qian W, Fang X, Shi Z, Li Y, Li S, Shan G, Kristiansen K, Li S, Yang H, Wang J, Wang J 2010 *De novo* assembly of human genomes with massively parallel short read sequencing. Genome Res 20:265–272. doi:10.1101/gr.097261.109. 20019144 PMC2813482

[5] Delcher AL, Bratke KA, Powers EC, Salzberg SL 2007 Identifying bacterial genes and endosymbiont DNA with Glimmer. Bioinformatics 23:673–679. doi:10.1093/bioinformatics/btm009. 17237039 PMC2387122

[6] Lagesen K, Hallin P, Rodland EA, Staerfeldt HH, Rognes T, Ussery DW 2007 RNAmmer: consistent and rapid annotation of ribosomal RNA genes. Nucleic Acids Res 35:3100–3108. doi:10.1093/nar/gkm160. 17452365 PMC1888812

[7] Schattner P, Brooks AN, Lowe TM 2005 The tRNAscan-SE, snoscan and snoGPS Web servers for the detection of tRNAs and snoRNAs. Nucleic Acids Res 33:W686–W689. doi:10.1093/nar/gki366. 15980563 PMC1160127

